# Strong coupling of collective intermolecular vibrations in organic materials at terahertz frequencies

**DOI:** 10.1038/s41467-019-11130-y

**Published:** 2019-07-19

**Authors:** Ran Damari, Omri Weinberg, Daniel Krotkov, Natalia Demina, Katherine Akulov, Adina Golombek, Tal Schwartz, Sharly Fleischer

**Affiliations:** School of Chemistry, Raymond and Beverly Sackler Faculty of Exact Sciences and Tel Aviv University Center for Light-Matter Interaction, Tel Aviv University, Tel Aviv, 6997801 Israel

**Keywords:** Polaritons, Chemical physics

## Abstract

Several years ago, strong coupling between electronic molecular transitions and photonic structures was shown to modify the electronic landscape of the molecules and affect their chemical behavior. Since then, this concept has evolved into a new field known as polaritonic chemistry. An important ingredient in the progress of this field was the demonstration of strong coupling with intra-molecular vibrations, which enabled the modification of processes occurring at the electronic ground-state. Here we demonstrate strong coupling with collective, inter-molecular vibrations occurring in organic materials in the low-terahertz region ($\lesssim$10^12^ Hz). Using a cavity filled with α-lactose molecules, we measure the temporal evolution and observe coherent Rabi oscillations, corresponding to a splitting of 68 GHz. These results take strong coupling into a new class of materials and processes, including skeletal polymer motions, protein dynamics, metal organic frameworks and other materials, in which collective, spatially extended degrees of freedom participate in the dynamics.

## Abstract (web summary)

Here, the authors demonstrate strong coupling between collective, terahertz inter-molecular vibrations of organic materials and a Fabry-Pérot cavity. These results extend the applicability of polaritonic chemistry to large-scale organic systems, such as biological macromolecules and polymer chains.

## Introduction

In the strong coupling regime, the interaction between light and matter overcomes all the incoherent and dissipative processes, which profoundly changes its nature. In this regime, the wavefunctions of the photons and the material excitations are coherently mixed to form hybrid light-matter quantum states known as polaritons^1^. This fascinating phenomenon has been observed in many different types of material systems, such as cold atoms^2–4^, excitons in semiconductors^5,6^, electronic spins in nitrogen-vacancy centers^7^, phonons in inorganic crystals^8–11^, and many others. Among these, strong coupling with organic molecules^12,13^ has seen an ever-increasing interest in recent years, both in conventional Fabry–Pérot cavity systems as well as in plasmonic structures^14^. Interestingly, the creation of the polaritonic wavefunctions under strong coupling and the modification of the energetic landscape of the molecules can have a significant influence on the physical and chemical properties of the molecules^1,15,16^, affecting the rates and yields of chemical reactions^17–22^, their emission properties^23–25^, electronic and excitonic transport^26–30^ and more. This new field, known as polaritonic chemistry, is currently under intense study, both experimentally and theoretically. While traditionally, strongly coupled organic systems involved the coupling of an optical resonance to electronic transitions in molecules (Frenkel excitons), recently, vibrational strong coupling has been introduced as a new paradigm^31–36^. In such systems, a particular intramolecular, optically active vibrational transition is coupled to a mid-infrared resonator, creating hybrid excitations termed “vibro-polaritons”. As has been demonstrated over the past few years, the creation of such vibro-polaritons allows the manipulation of molecular processes occurring at the electronic ground-state, by targeting a specific intra-molecular bond and coupling its associated vibration to an optical mode^18,37,38^.

A different class of vibrational modes in organic materials are inter-molecular vibrations, which are particularly relevant in large molecular structures such as organic crystalline materials, polymers, and proteins. These vibrations, which typically lie in the terahertz frequency regime (10^11^–10^13^ Hz, or several tens of cm^−1^), correspond to the concerted motion of the unit cells, one with respect to another (rather than internal vibrations of atoms within each molecule). Moreover, these collective vibrations are extended over many inter-molecular bonds. Here, we demonstrate strong coupling of collective vibrations in ensembles of organic α-lactose molecules, occurring at 0.53 THz. Unlike the previously studied vibrational strong coupling, here the cavity mode is coupled to the inter-molecular vibration extended over the crystallites, i.e. the oscillatory motion of the hydrogen-bonded molecules with respect to one another. We observe the Rabi-splitting typical of strong coupling and coherent Rabi-oscillations at room temperature, despite the fact that the energy of the collective vibrational transition ($\hbar \nu _{{\mathrm{vib}}}$~2 meV), as well as the light-matter interaction strength, are much lower than *k*_B_T (~25 meV). Our results extend the applicability of polaritonic chemistry to a plethora of large-scale organic systems, such as biological macromolecules^39^, polymer chains^40^, energetic materials with low lying collective vibrations^41^, skeletal motions in metal organic frameworks (MOFs)^42^, and many others.

## Results

### Hybrid cavity-lactose system

**Fig. 1:**
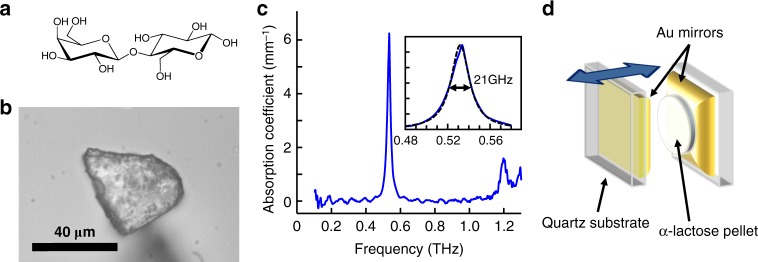
α-Lactose molecules in a tunable THz cavity**. a** Chemical structure of α-lactose. **b** A microscope image of an α-lactose crystallite. **c** Absorption coefficient of the α-lactose pellet obtained from the THz absorption measurement. The inset shows the fit of the measured absorption peak (blue line) to a lorentzian line-shape (black dashed line). **d** A sketch of the open THz cavity used in the experiments

Lactose is a disaccharide composed of galactose and glucose, and it is one of the primary ingredients of milk. In this study we use α-lactose monohydrate, which is one of the anomers formed upon the crystallization of lactose, with the chemical structure shown in Fig. 1a. The α-lactose powder used in this study (as purchased from Sigma-Aldrich) is comprised of small, polycrystalline particles, a few tens of microns in size, as shown in Fig. 1b. To measure its THz absorption spectrum, we prepared a ~1.3-mm-thick pellet of pristine α-lactose using a pressing die (see Methods section), and measured its absorption spectrum using terahertz time-domain spectroscopy (THz-TDS). From this measurement we obtain the frequency-dependent absorption coefficient, which is given by $\alpha \left( \nu \right) = {\mathrm{{ln}}}\left( {10} \right)A/l$ with *A* being the absorption spectrum and *l* the pellet thickness. The result is presented in Fig. 1c, showing a sharp absorption peak at 0.53 THz (17.7 cm^−1^) with a width of 21 GHz full-width half-maximum (FWHM). This absorption line corresponds to a collective, intermolecular vibration in the molecular crystal, in which the molecules move with respect to each other as a rigid body^43–45^. An additional weaker absorption peak is observed within our usable THz bandwidth, at 1.2 THz. In order to demonstrate strong coupling of the collective vibrational mode at 0.53 THz, we utilized the open-cavity geometry^46^ depicted in Fig. 1d (see Methods section for further details). The cavity is composed of two Au mirrors prepared by sputtering ~6 nm Au layers on 1-mm-thick quartz substrates. The reflection amplitude of the mirrors was measured to be ~90% for the THz field (81% reflectivity). In the open cavity geometry, one of the mirrors is fixed, while the other is mounted on a computer-controlled translation stage, parallel to the fixed mirror, such that the cavity length *d* can be varied continuously.

### Terahertz spectroscopy measurements

**Fig. 2:**
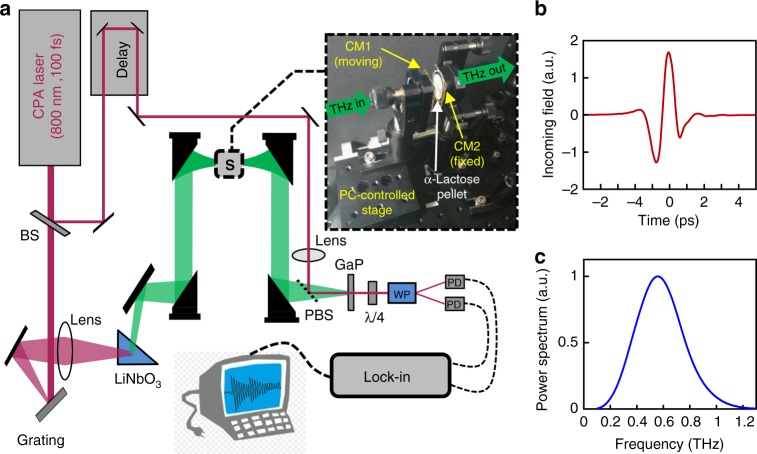
Time-domain THz spectrometer. **a** Schematic sketch of the optical setup. The inset shows a photograph of the open cavity, which is formed by a moveable mirror (CM1) and a fixed mirror (CM2). **b** The time-resolved single-cycle THz field used in this work and **c** its power spectrum, obtained by the Fourier transform of the data in **b**

The measurements were performed using a home-built, time-domain terahertz spectrometer, shown in the schematic diagram in Fig. 2a. In a typical measurement, an ultrashort laser pulse (100 fs pulse duration, 800 nm central wavelength) from a Ti:Sapphire chirped pulse amplifier (Legend Duo, Coherent Inc.) is split to form a strong optical beam for THz generation and a weak readout pulse for time-resolved electro-optic sampling of the THz field^47,48^. A single-cycle THz pulse is generated via tilted pulse-front optical rectification in LiNbO_3_ (LN)^49^ and focused through the sample (S), which is placed at the focal plane of a 4-f setup composed of two off-axis parabolic reflectors. The THz field and the readout pulse are combined by a pellicle beam-splitter (PBS) and focused at the electro-optic detection crystal (Gallium Phosphate, GaP), following which the probe beam is analyzed for its differential polarization changes (by splitting the two polarizations with a Wollaston Prism [WP] and a pair of photodiodes [PD]). The signals are detected using a lock-in amplifier (Stanford Research Systems, SR830) and recorded by a desktop PC. The peak field strength of the THz pulse is ~50 kV/cm. The entire system is purged with dry air (relative humidity <4%) to eliminate THz absorption by the water vapor in the ambient lab atmosphere (water absorption lines of 0.56, 0.75, and 0.98 THz are clearly observed in our THz spectrum and are completely removed when the system is purged with dry air). Also shown in Fig. 2 are the measured electric field of the generated THz pulse (Fig. 2b) and its calculated power spectrum (Fig. 2c). As seen, the input field is indeed a single-cycle pulse, centered around 0.6 THz with a usable spectrum covering the 0.1–1.2 THz range.

**Fig. 3:**
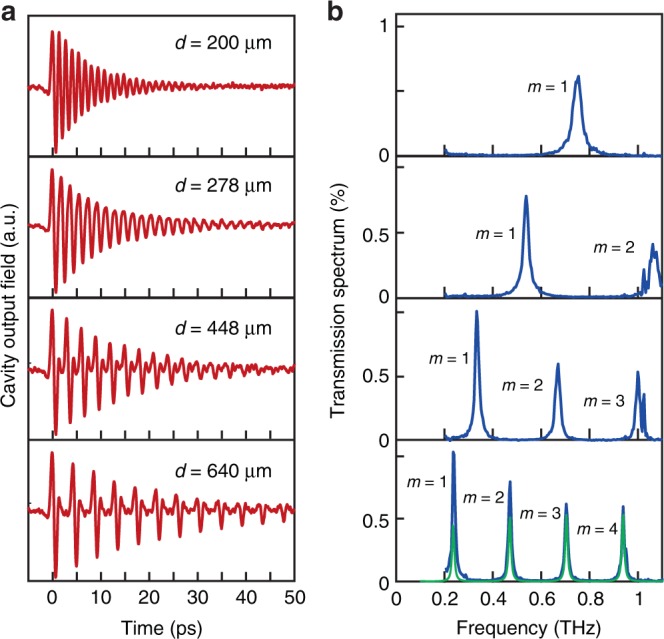
Time-domain THz spectroscopy of an empty cavity. **a** Time-resolved THz signal measured at the output of the empty cavity with different distances, *d*, between the mirrors, showing the exponentially decaying oscillations of the field exiting the cavity. **b** Transmission power spectra of the cavity with increasing cavity lengths, showing the gradual progression of the empty-cavity resonant modes. The green line shows the transmission spectrum obtained by *T*-matrix calculations

First, we characterized the response of the empty cavity, i.e., when the gap between the mirrors contains only dry air. Note that in such time-domain experiments, parasitic reflections within the THz-TDS spectrometer result in spurious signals which enter the measurements. As detailed in Supplementary Note 1, we deconvolute the raw signal from the instrument response function to eliminate these distortions^50,51^. The results of the THz-TDS (following deconvolution) are presented in Fig. 3a, showing the time-domain signal of the field exiting the cavity, for several different cavity length values (at normal incidence). As can be seen, when the single-cycle THz pulse passes through the cavity, it is stretched to an exponentially decaying oscillatory signal, as expected for a resonant cavity with a finite lifetime. The transmission spectrum of the cavity can then be calculated by taking the ratio between the power spectra of the (deconvoluted) transmitted signals shown in Fig. 3a and dividing them by the input pulse power spectrum, shown in Fig. 2c. These transmission spectra, calculated for the different cavity lengths, are shown in Fig. 3b. As can be seen, the resonant Fabry–Pérot cavity modes are clearly visible, with their frequencies obeying the relation $f_m = \frac{c}{{2d}}m$, where *c* is the speed of light, *d* is the cavity length (the distance between the mirrors), and *m* is the mode number (assigned in Fig. 3b). Specifically, for a cavity length of 640 μm, for which the second-order mode is close to the α-lactose absorption line, the resonant modes have a transmission peak of 0.5–1% and a linewidth of ~14 GHz, matching the calculated finesse for mirrors with reflectivity of 81%. In addition, we performed transfer-matrix calculations (see Methods section) for the 640 μm cavity to simulate the spectral response of the cavity (solid green line in Fig. 3b), which agree with the experimental measurement.

**Fig. 4:**
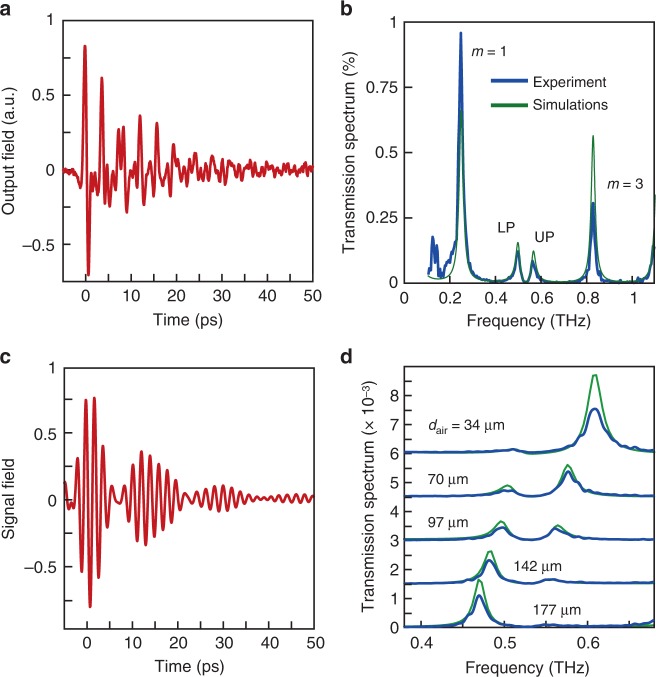
Strong coupling of collective THz vibrations in α-lactose. **a** Measured time-domain output signal from the cavity with the α-lactose pellet, with its length chosen such that the second cavity resonance (*m* = 2) is at 0.53 THz. **b** Transmission spectrum of the cavity with α-lactose with a total cavity length of 350 μm, obtained from the experimentally measured time domain signal in Fig. 4a (blue line) and by T-matrix calculations (green line). The strong coupling between the collective vibrations and the cavity results in a spectral splitting that indicates the formation of hybrid vibro-polariton states. **c** Spectrally filtered output signal from the cavity, showing the Rabi-oscillations of the coupled system in the time-domain. **d** Selected cavity transmission spectra in the vicinity of the α-lactose absorption peak (0.53 THz), measured with different air gap thicknesses *d*_air_ and α-lactose pellet thickness of *d*_αL_ = 250 μm (blue lines), compared to T-matrix calculations (green lines)

Next, we examined the response of the cavity with the α-lactose pellet placed between the mirrors. We prepared an α-lactose pellet of 250 μm in thickness, attached it to the fixed mirror and adjusted the total cavity length to ~350 μm. Under such conditions, the effective optical length of the cavity (given by $d_{{\mathrm{opt}}} = d_{{\mathrm{\alpha L}}}n_{{\mathrm{\alpha L}}} + d_{{\mathrm{air}}}$ with *d*_αL_ being the pellet thickness, *n*_αL_ = 1.8 the background refractive index of α-lactose^52^, and *d*_air_~100 μm is the thickness of the air-gap) is ~550 μm, such that the second-order cavity mode is resonant with the collective vibrational mode of the α-lactose at 0.53 THz. We note that we chose to target the second-order mode of the cavity since tuning the first cavity mode to 0.53 THz would require a thinner α-lactose pellet (~150 μm), which results in fragility and inhomogeneity of the pellet. The time-resolved THz field exiting the cavity is presented in Fig. 4a. We observe a similar exponentially decaying oscillation, as in the empty cavities, but here the signal is modulated by a periodic envelope. This periodic modulation corresponds to Rabi-oscillations in the cavity—an excitation that is shared by both the photonic cavity and the inter-molecular vibration of the α-lactose, serving as an indication of the strong coupling between those two entities^1,2,53^. The transmission spectrum of the α-lactose cavity, obtained using the Fourier transform of the signal in Fig. 4a, is shown in Fig. 4b (blue solid line). Furthermore, by fitting the results to transfer-matrix calculations (using the experimentally measured refractive index of α-lactose^52^), shown by the green solid line, we obtain a thickness of *d*_αL_ = 250 μm for the α-lactose pellet and *d*_air_ = 97 μm for the air gap thickness. As can be seen in both the experimental measurement and the simulations, the hybrid cavity/α-lactose system exhibits a clear splitting in the spectral response around the collective vibration frequency and the formation of two THz vibro-polariton states, at 0.50 and 0.56 THz, indicating once again that the hybrid system is indeed within the strong coupling regime. In addition to the polaritonic modes, the first (*m* = 1)- and third (*m* = 3)-order cavity modes at 0.25 and 0.83 THz are also located within the bandwidth of the input pulse. Therefore, the single-cycle pulse excites a coherent superposition of the polaritonic modes as well as the non-coupled cavity modes, which gives rise to the seemingly irregular dynamics seen in the time domain (Fig. 4a). To illustrate this, we numerically filter the time-domain signal by a band-pass filter, leaving only frequencies within the range of 0.38–0.68 THz. The filtered time-domain signal is presented in Fig. 4c. As seen, the Rabi-oscillations of the coupled system are clearly observed, demonstrating the reversible and coherent light-matter interaction taking place in the system. Interestingly, Rabi oscillations occurring under strong coupling of molecular excitons and plasmonic structures were previously observed by probing the excited state population, using ultrafast pump-probe spectroscopy^53^. However, here we are able to observe the Rabi-oscillations in the emitted field directly, including its oscillating phase. It is important to note that the Rabi splitting observed in our experiment (as also in other polaritonic systems) is not induced by the THz pulse itself. Instead, the THz field merely probes the coupled states of the system, which are formed by the interaction of the molecular vibrations with the cavity mode^1,14^. This is also evident by the fact that the spectral response of the coupled cavity (shown in Fig. 4b) does not change when the intensity of the THz pulse is varied (see Supplementary Note 2).

### Vibro-polariton dispersion

**Fig. 5:**
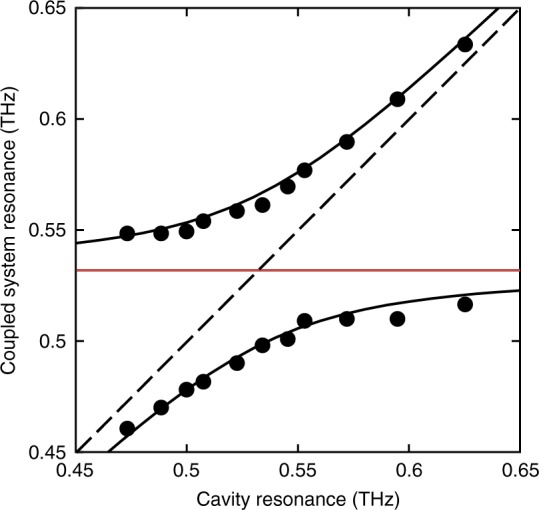
THz vibro-polariton branches of strongly coupled α-lactose. The circles correspond to the measured polariton peaks, which were obtained by scanning the cavity resonance across the α-lactose absorption (marked by the horizontal red line). The dashed black line marks the empty cavity resonance while the solid black curves show the result of the coupled oscillator model (Eq. (1)) fitted to the measured data

Next, we varied the position of the moveable mirror, repeated the measurement, and calculated the spectral response as shown in Fig. 4b, for several different cavity lengths. As shown in Fig. 4d (blue lines), when the cavity is detuned from the vibrational absorption peak, the frequencies of the polaritonic modes are shifted relative to the resonant case, and the relative strengths of their transmission peaks also changes. As expected, in the time domain (see Supplementary Fig. 5) these features are manifested by a lower modulation depth of the Rabi oscillations and a shorter Rabi cycle for the oscillating envelope, as compared to Fig. 4c. Also shown in Fig. 4d are the simulated transfer-matrix results for the coupled system (green lines, see Methods section for further details). In our simulations, we fix all the parameters except for the thickness of the air-gap between the pellet surface and the moveable mirror, which is extracted by fitting the simulated transmission spectrum to the experimental data (note that the transfer-matrix formalism provides the linear spectral response of the cavity, thus the excellent agreement between the experimental and the simulated transmission data further indicates that the THz field only serves as a probe for the resonances of the coupled system and does not govern the strong-coupling effect, as also verified experimentally in Supplementary Note 2). Using these simulations, we can extract the second-order cavity resonance for each value of the cavity thickness, by removing the contribution of the vibrational resonance to the refractive index of the α-lactose, only taking into account its background index of refraction. Finally, we use these results to plot the dispersion of the hybrid molecular/cavity system, i.e. the measured vibro-polariton frequencies as a function of the cavity resonance frequency. As seen in Fig. 5, the dispersion shows the formation of the characteristic polariton branches around the absorption frequency of the α-lactose collective vibration. We fit these measurements to the dispersion resulting from the coupled-oscillator model, which gives the real part of the resonance frequencies of the coupled system as^54^

1 $$\nu _ \pm = \frac{{\nu _{\mathrm{c}} + \nu _{{\mathrm{vib}}}}}{2} \pm \sqrt {\left( {V/h} \right)^2 + \frac{1}{4}\left[ {\left( {\nu _{\mathrm{c}} - \nu _{{\mathrm{vib}}}} \right) - \frac{i}{2}\left( {\gamma _{\mathrm{c}} - \gamma _{{\mathrm{vib}}}} \right)} \right]^2},$$

where *V* is the dipolar light-matter interaction energy (see Methods section for further details), *h* is Planck’s constant, $\nu _{{\mathrm{vib}}} = 0.53\, {\mathrm{{THz}}}$ is the collective vibration frequency, *v*_c_ is the cavity frequency (of the second-order mode), and *γ*_vib_ = 21 GHz and *γ*_c_ = 14 GHz are their FWHM linewidths. At resonance (*v*_c_ = *v*_vib_), the difference between the two polaritonic frequencies gives the Rabi frequency of the couple system $\nu _{\mathrm{R}} = 2\sqrt {\left( {V/h} \right)^2 - \frac{1}{{16}}\left( {\gamma _{\mathrm{c}} - \gamma _{{\mathrm{vib}}}} \right)^2}$. By fitting Eq. (1) to the measured data, we obtain a Rabi frequency value of *v*_R_ = 68 GHz, which also reflects the coupling energy *V* = *hv*_R_ under the experimental conditions (since $\gamma _{\mathrm{c}} - \gamma _{{\mathrm{vib}}} \ll \nu _{\mathrm{R}}$). This value satisfies the condition $\nu _{\mathrm{R}} > \frac{{\gamma _{\mathrm{c}} - \gamma _{{\mathrm{vib}}}}}{2}$, confirming that our system is indeed within the coherent, strong coupling regime^14^. Moreover, this Rabi splitting value is about 13% of the bare vibration frequency, placing this system close to the ultrastrong coupling regime.

## Discussion

We have demonstrated the strong-coupling of the collective vibration of α-lactose crystallites and a Fabry-Pérot cavity in the low-THz frequency region (0.53 THz), and observed a Rabi-splitting of ~13% of the fundamental frequency. Moreover, we have observed the coherent vacuum Rabi-oscillations taking place in the coupled system, taking advantage of the ability to perform time-domain and phase-sensitive measurements of the THz field. Interestingly, since the measurements are performed at room temperature, the energy of the collective vibration *hv*_vib_ = 2.2 meV and the Rabi-splitting energy *hv*_R_ = 0.28 meV are both lower than *k*_B_*T*. This places our system in a very different regime than all previously studied organic strong-coupled systems, and expected to result in a different coupling to the surrounding thermal bath and therefore different relaxation dynamics^55^.

Finally, in contrast to previous works that studied vibrational strong coupling with intra-molecular vibrations or phonons in inorganic crystals, where covalent bonds were strongly coupled to the photonic modes, here we strongly couple the hydrogen bonds by which the molecules are held together. Such weak hydrogen bonds, whose vibrational frequencies often reside in the THz range^39^, are central to supramolecular chemistry and biological functionality. In the context of polaritonic chemistry, the ability to affect these weak bonds by modifying their collective vibrational modes may be used to judiciously control chemical and biological processes that depend on extended degrees of freedom. For example, collective THz vibrational excitations are involved in the conformational changes and binding events in proteins^56–58^, and therefore modifying the natural vibrational modes by strong coupling is expected to alter the biological functionality of proteins, in a similar manner to heavy element substitution^36^. Furthermore, many biological processes are governed by the collective low-frequency vibrations of the surrounding hydrogen-bonded water network^59^, which may also be affected by strong coupling. Going beyond biology, the mechanical and morphological properties of polymers and organic crystals are intimately linked to THz-active vibrations of the inter-molecular hydrogen bonds^42,60,61^, and in various chemical processes, such as detonation in energetic materials, mechanical energy on the macroscopic scale is funneled through collective THz modes into intra-molecular bonds, to drive the chemical reaction^62^. In such systems, as well as others, strong coupling between collective THz vibrations and an optical resonator may be used to manipulate the material properties and reaction pathways.

The relatively easy fabrication of the open-mirror-cavity demonstrated here, together with the direct measurement of the amplitude and phase of the electric field in time-domain THz spectroscopy, provide an extended test-bed for studying the very basic underlying physics of the strong-coupling phenomenon. Furthermore, the relatively large (tens to hundreds of μm) length-scale of the THz cavity makes it accessible to additional stimulations, such as optical excitations, that may alter the molecular structure, as well as to structural patterning of the sample to manipulate the light-matter interaction within cavity.

## Methods

### Open cavity configuration

The variable-length open cavity used in this work (see Fig. 2) is composed of a moveable mirror (CM1) and a fixed mirror (CM2). Both mirrors were produced by sputtering a thin layer (6 nm) of gold on a quartz substrate (1 mm thickness), resulting in a transmission amplitude of 90% across the whole usable bandwidth with no apparent spectral dependence. CM1 is mounted on a computer-controlled single-axis stage, with micrometer resolution (<2 μm repeatability). By moving CM1 with respect to CM2 we control the length of the cavity and corresponding resonance frequency. CM1 and CM2 are set parallel to each other by coinciding the multiple reflections of a green diode laser from the mirrors at the far field. The α-lactose sample (white, round pellet) was prepared by placing 0.1 g of α-Lactose powder in a pressing die (20 mm diameter) at a pressure of 220 kN for 10 min, which yielded a ~250 μm pellet. The pellet was then glued onto CM2 at a few points around its circumference.

### Transfer matrix calculations

The simulated transmission spectra of the cavity were calculated using the transfer-matrix formalism, which provides the linear transmission spectrum of the cavity (with and without the α-lactose) by solving the wave propagation through the multilayer structure^35,63^. In these simulations, we used the experimentally measured refractive index of gold^64^ to model the cavity mirrors and adjusted the thickness of the mirrors to match the measured reflectivity of 81%. We note that the fitted thickness of the mirrors was found to be 1.5 nm, which is lower than the actual thickness of 6 nm. This is most probably due to the fact that at such low thicknesses the sputtered metal film is not continuous, but rather composed of small Au islands. The interaction with the molecules can be modeled classically by including their frequency-dependent complex dielectric function in the transfer-matrix simulations^14,35,54,65^. Within our usable THz range, the dielectric function of the α-lactose pellet is accurately described by a Lorentz–Drude model with contributions from three different vibrational transitions^52^

2 $${\it{\epsilon }}_{{\mathrm{\alpha L}}}\left( \nu \right) = {\it{\epsilon }}_0\left[ {{\it{\epsilon }}_\infty + \mathop {\sum }\limits_{i = 1}^3 \frac{{\nu _{{\mathrm{p}},i}^2}}{{\nu _i^2 - \nu ^2 - i\gamma _i\nu }}} \right],$$

where *ε*_0_ is the vacuum permittivity, *ε*_0_ = 3.2 is the (relative) background dielectric constant, *v*_*i*_ = 0.53, 1.195, and 1.37 THz are the vibrational frequencies, *γ*_*i*_ = 21, 44, and 58 GHz are the linewidths, and *v*_p,i_ = 0.123, 0.072, and 0.253 THz are the corresponding plasma frequencies.

### Microscopic estimation of the Rabi frequency

The vacuum Rabi frequency is related to the collective dipolar coupling between the optical transition in the molecules and the field contributed by a single photon in the cavity^1,14^, given by $V = d\sqrt {\frac{{h\nu }}{{2{\it{\epsilon }}_0{\it{\epsilon }}_\infty V_{\mathrm{c}}}}N}$ with *d* being the transition dipole element of each molecule, *h* is the Planck constant, *v* is the cavity resonance frequency (taken as equal to the vibrational transition frequency of 0.53 THz), *ε*_∞_ is the background dielectric constant inside the cavity, *V*_c_ is the cavity mode volume, and *N* the number of molecules (or number of resonant vibrating bonds, in the case of vibrational strong coupling). Under resonant conditions (Fig. 4a–c) the lactose pellet almost fills the entire gap between the mirrors. For metallic cavities under such conditions, the volume occupied by the molecules can be approximately taken as the mode volume^66^, such that the ratio *N*/*V*_c_ can be roughly taken as the molecular concentration. With a molar weight of 342 g mol^−1^ and a density of 1.52 g cm^−3^, this concentration is $n = 2.6 \times 10^{21}\, {\mathrm{{cm}}^-3}$. In our system, the cavity mode is coupled to a collective vibration, which is extended across many molecules and therefore the coupling needs to be described in terms of a collective transition dipole operator, in a similar manner to ref. ^35^. However, one may define an effective molecular transition dipole, which can be extracted from the absorption spectrum shown in Fig. 1c while treating the molecules as individual absorbers. Using the relation $d^2 = \frac{{3{\it{\epsilon }}_0ch}}{{2\pi ^2\nu }}{\mathrm{\Delta }}\nu \sigma _{{\mathrm{abs}}}$ (where *ε*_0_ is the dielectric constant of vacuum, *c* the speed of light, *v* the transition frequency, Δ*v* the absorption linewidth, and σ_abs_ is the absorption cross section^67^) and taking $\sigma _{{\mathrm{abs}}} = \alpha /n$ with the measured absorption coefficient *α* = 6.3 mm^−1^ at the 0.53 THz line, we obtain a transition dipole element of *d* = 0.06 Debye. Using this value and *ε*_∞_ = 3.2, the expected coupling energy is *V* = 0.16 meV. This corresponds to a Rabi frequency of 2 *V*/*h* = 76 GHz, in very good agreement with our experimental observation.

## Supplementary information

Supplementary Information

## Data Availability

The data that support the findings of this study are available from the corresponding author upon reasonable request.
