# Supplementary material for: Strong coupling of collective intermolecular vibrations in organic materials at terahertz frequencies
Source: Nat Commun. 2019 Jul 19;10:3248. doi: 10.1038/s41467-019-11130-y (PMC6642260; doi:10.1038/s41467-019-11130-y)
Supplement: Supplementary file 1 — Supplementary Information [file 41467_2019_11130_MOESM1_ESM.pdf]

Supplementary Information

Strong Coupling of Collective Intermolecular Vibrations in Organic Materials at Terahertz Frequencies

Ran Damari, Omri Weinberg, Daniel Krotkov, Natalia Demina, Katherine Akulov, Adina Golombek, Tal Schwartz and Sharly Fleischer**

School of Chemistry, Raymond and Beverly Sackler Faculty of Exact Sciences and Tel Aviv University Center for Light-Matter Interaction, Tel Aviv University, Tel Aviv 6997801, Israel.

***Corresponding authors: talschwartz@tau.ac.il, sharlyf@tauex.tau.ac.il**

Supplementary Note 1: Signal processing and deconvolution scheme

Time-resolved electro-optic sampling measurements suffer from various satellite signals that accompany the signals of interest, particularly in such long-time measurements as used in our experiments (~100ps long). Those artifact signals originate from various reflections of both the THz-field and the optical read-out pulse in the electro-optic detection crystal (typically GaP/ZnTe). Moreover, in our case, additional internal reflections within the 1 mm-thick quartz substrates used for the mirrors CM1 and CM2 (between the Au layers and the quartz-air interface) are also observed in the raw data that is measured.

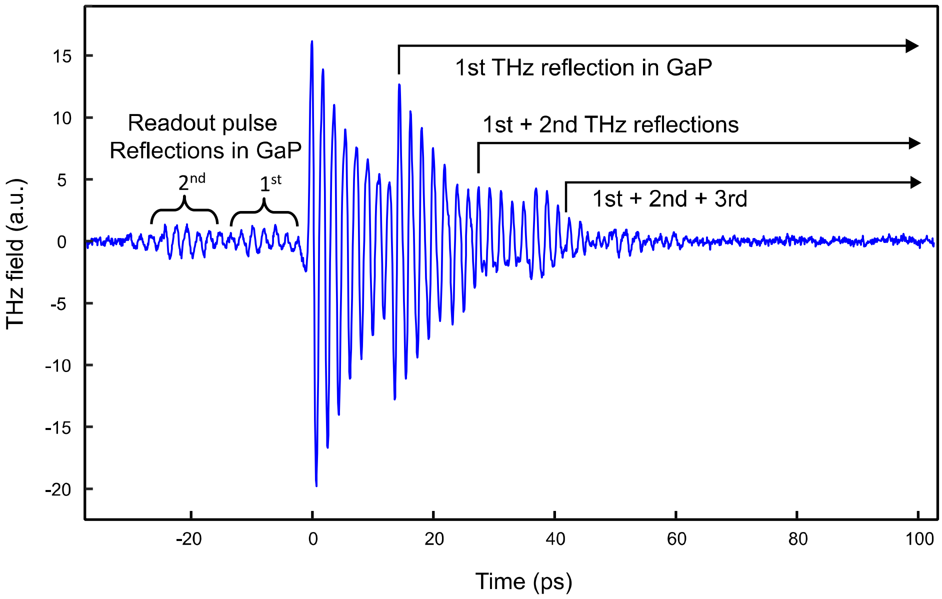

Supplementary Figure 1. Raw THz signal (as recorded) transmitted through an empty cavity (without α-lactose). The main artifact contributions are marked in the figure and arise from multiple reflections of both the THz field and the optical readout pulse in the electro-optic sampling crystal (GaP). Since the "real" oscillatory cavity response extends beyond the time of the first THz reflection (the cavity life-time >12ps), the artifact reflections overlap with the signal of interest.

While the multiple reflections of the THz field in the GaP sampling crystal result in repetitive signals which extend toward the positive-time direction of our measurement (namely following the "real" THz field, with periodicity of ~12 ps for our 0.5 mm GaP crystal), the reflections of the optical readout pulse are manifested as signals at negative time. As shown in Supplementary Figure 1, the actual signals of interest are clearly also affected by these multiple reflections and result in artifact modulations in the time-domain signal (and in the frequency domain following Fourier transformation) that may obscure the pure response of the cavity.

To overcome this difficulty we implement a deconvolution scheme by which the signal of interest can be extracted from the raw signal[1,2]. First, we obtain the transfer function of the TD-THz spectrometer, $h(t)$, by performing a reference measurement of a thin empty cavity (~65 μm), such

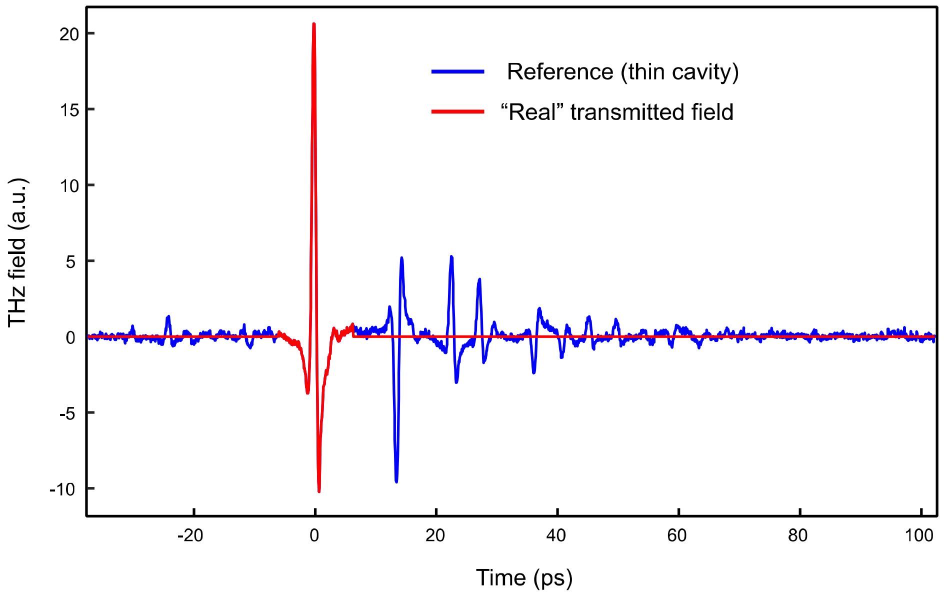

Supplementary Figure 2. Empty reference cavity signal ($Ref(t)$, blue curve) and the "real" single-cycle THz pulse ($p(t)$, red curve).

that even its lowest resonant frequency ($c/2d$=2.3 THz) lies beyond our usable THz frequency span (0.1-1.2 THz), and its transmission spectrum, determined by the (weak) transmission of the individual mirrors, is uniform across the whole usable spectrum. The blue curve in Supplementary Figure 2 depicts the raw data obtained for the reference cavity ($ref(t)$), which is the result of a convolution of the real single-cycle THz field ($p(t)$, red curve) with the complicated transfer function of our setup, including the multiple reflections in the quartz substrates. Thus, the transfer function of the system can be extracted in the frequency domain, using the relation

$$H(\nu) = \frac{Ref(\nu)}{P(\nu)} \tag{1}$$

with $P(\nu) = \mathcal{F}\{p(t)\}$ and $Ref(\nu) = \mathcal{F}\{ref(t)\}$ being the Fourier transforms of the real transmitted pulse and the measured reference, respectively.

In a similar manner, for each one of the measured signals of the cavity (with and without the α-lactose), the raw (measured) signal $s_{\mathrm{M}}(t)$ is given by the convolution of the real signal $s(\mathrm{t})$ with the transfer function $h(t)$. Therefore, the real signal transmitted through the cavity can be obtained using

$$s(\mathrm{t}) = \mathcal{F}^{-1}\left\{\frac{\mathrm{S_M}(\nu)}{H(\nu)}\right\}. \tag{2}$$

The result of such deconvolution procedure for an empty cavity is shown in Supplementary Figure 3.

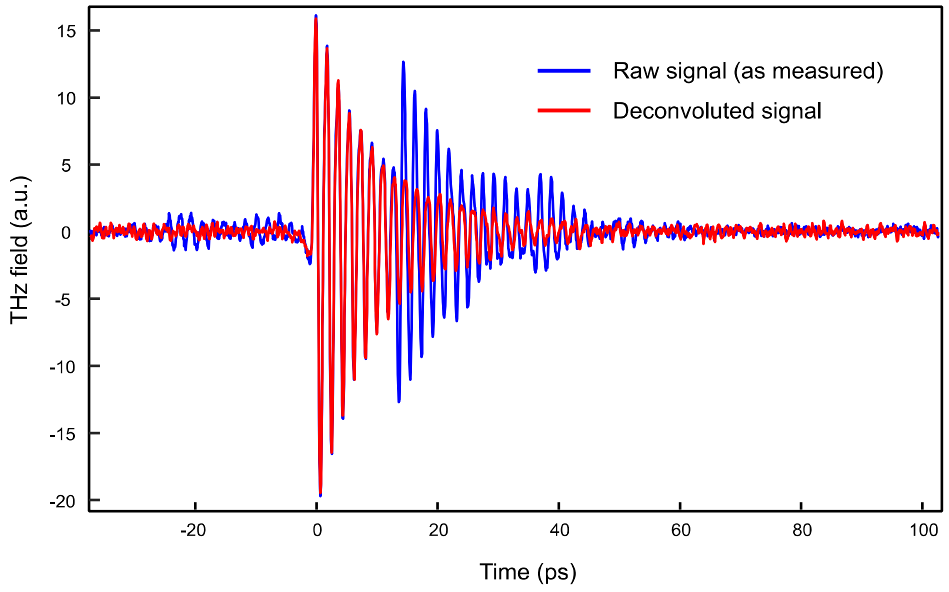

Supplementary Figure 3. Raw signal (blue curve) from an empty cavity (d=278 μm) and its extracted signal (red curve) following deconvolution with $h(t)$. Note that the oscillatory signal of the cavity at the t >12 ps region is clearly observed and the large artifact reflections have been removed.

Supplementary Note 2: Intensity independence of the Rabi splitting

In order to verify that the Rabi splitting is not induced by the THz probe pulse, we repeated the measurements on a cavity with an α-lactose pellet under similar conditions as in Figure 4b with several different intensities of the THz pulse, which was achieved by varying the intensity of the pump beam which generated the THz field. Supplementary Figure 4 shows the power spectra of the transmitted THz fields (with peak field strengths of 50, 30 and 17 kV/cm for the input pulses). For all three cases the frequencies of the polaritonic resonances, and hence also the Rabi splitting values, do

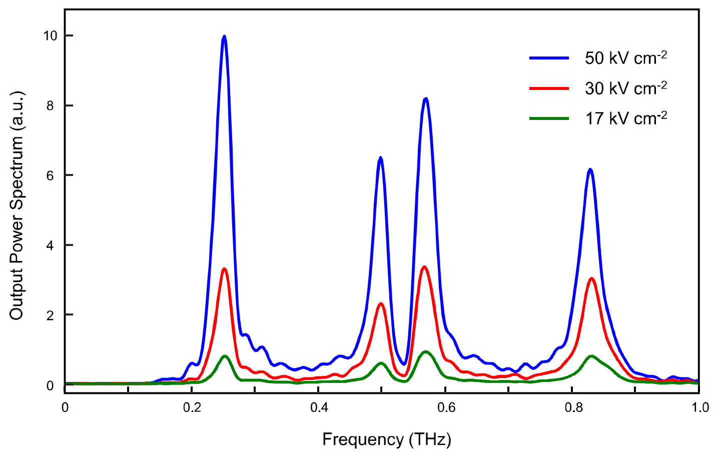

Supplementary Figure 4. Power spectra at the output of an α-lactose cavity measured with different input pulse intensities.

not vary with the pulse intensity, confirming that the THz pulse only acts as a probe for the linear modes of the coupled system. Notice that the sample used in these measurements is slightly different than the one used for Figures 3-5, however, all three measurements shown in Supplementary Figure 4 were conducted with the same sample.

Supplementary Figure 5: Time-domain signals for detuned cavities

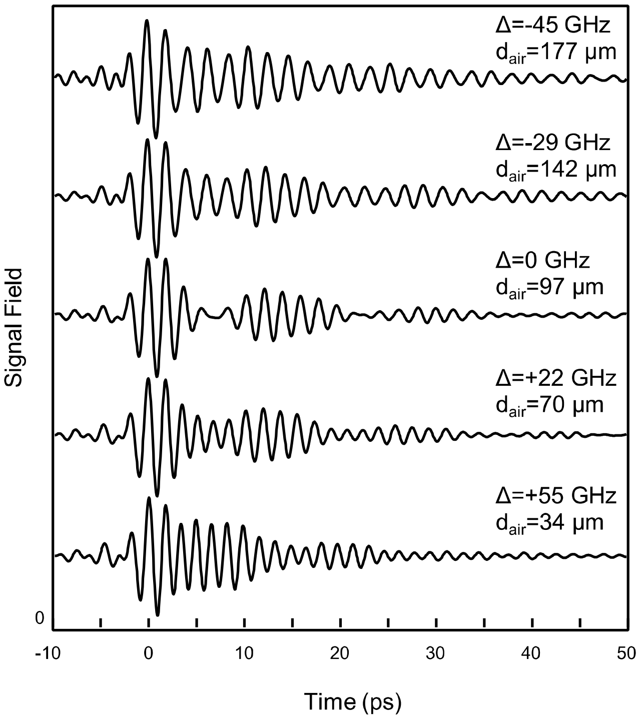

Supplementary Figure 5. Filtered time-domain signals (obtained as in Figure 3c) for the detuned cavities shown in Figure 4d. $\Delta = \nu_c - \nu_{vib}$ is the detuning between the cavity resonance and the α-lactose absorption peak.

Supplementary References

1. Damari, R., Kallush, S. & Fleischer, S. Rotational Control of Asymmetric Molecules: Dipole- versus Polarizability-Driven Rotational Dynamics. *Phys. Rev. Lett.* **117**, 103001 (2016).

2. Damari, R., Rosenberg, D. & Fleischer, S. Coherent Radiative Decay of Molecular Rotations: A Comparative Study of Terahertz-Oriented versus Optically Aligned Molecular Ensembles. *Phys. Rev. Lett.* **119**, 033002 (2017).
